# Determination of bioactive compounds, antioxidant and anticancer activities of Tuckeroo (*Cupaniopsis anacardioides*) fruits

**DOI:** 10.1007/s13205-022-03314-z

**Published:** 2022-09-03

**Authors:** Ngoc Minh Quynh Pham, Quan V. Vuong, Jennette A. Sakoff, Michael C. Bowyer, Van Anh Le, Christopher J. Scarlett

**Affiliations:** 1 Department of Applied Sciences, School of Environmental and Life Sciences, University of Newcastle, Brush Rd, Ourimbah, NSW 2258 Australia; 2 Department of Biotechnology, Institute of Biotechnology and Environment, Nha Trang University, Nha Trang, Vietnam; 3 Department of Medical Oncology, Calvary Mater Newcastle Hospital, Waratah, NSW 2298 Australia

**Keywords:** Tuckeroo, Fruits, Phytochemical, Antioxidant, Anticancer, Cytotoxic activities

## Abstract

This study aimed to determine the phytochemical, antioxidant, and anticancer activities of the crude extract and its fractions of *Cupaniopsis anacardioides*. The results showed that total phenolic content (TPC), their secondary metabolites (flavonoids—TFC; proanthocyanidins—TPro), and antioxidant activity were significantly different between the crude extract and its fractions. The butanol fraction (F3) had the highest levels of TPC, TFC, and TPro, followed by the crude extract, aqueous fraction (F4), dichloromethyl fraction (F2), and hexane fraction (F1). High-Pressure Liquid Chromatography (HPLC) analysis revealed 14 major bioactive compounds were identified in the *C. anacardioides* extract. Further analysis showed F3 fraction contained the highest levels of the major bioactive compounds, while F1 fraction had the lowest. A similar pattern was observed for antioxidant activities. The crude extract, F3 and F4 fractions were further tested for cytotoxicity against 10 cancer cell lines, including HT29 (colon); U87, SJG2 (glioblastoma); MCF-7 (Breast); A2780 (ovarian); H460 (lung); A431 (skin); Du145 (prostate); BE2-C (neuroblastoma); MIA PaCa-2 (pancreas); and one non-tumour-derived normal breast cell line (MCF10A). Except for Du145 (prostate), the crude extract, F3 and F4 fractions inhibited the cancer cell lines at 100 µg/mL, with F3 possessing greater activity against these cancer cell lines. Future studies are recommended to isolate and identify the major bioactive compounds of the F3 fraction, and further tested their impact against cancer cell lines. This could identify the potential of anticancer agents from *C. anacardioides*.

## Introduction

Plant-derived phytochemicals have been used as a natural medicine for the treatment of diseases for thousands of years (Hartwell 1982; Tabor 2002). Approximately 60% of commercial pharmaceutical products are related to various plant species, and are used by ~ 80% of rural populations worldwide. This demonstrates that plant biological compounds are indispensable agents in the prevention and treatment of human diseases (Cragg and Newman 2005). However, only 10% of the ~ 250,000 plant species have been investigated for their medicinal potential (Q. Vuong et al. 2014). Therefore, seeking, screening, and identifying such phytochemicals are essential for exploring a novel and an effective therapeutic agent. In particular, cancer diseases are known as largely untreatable diseases due to the toxicity of modern chemotherapy and cancer cell resistance to anticancer agents (Duell et al. 2012; Hidalgo 2010; Li and Leung 2014; Neoptolemos et al. 2004; Scarlett and Vuong 2015). Therefore, there is an urgent need to identify the effective prevention and treatments methods for these diseases.

The Tuckeroo (*Cupaniopsis anacardioides*) is one of Australian nature plant, its fruit performed in eye-catching colour, orange, was used as a natural food source by Aboriginal people in Australia for hundreds of decades ago (Everitt and Alaniz 1981). Previous study (Pham et al. 2017) was indicated Tuckeroo fruits possess a high level of phenolics compounds and strong antioxidant capacity that could be a valuable promising for anticancer treatments.

This study aimed to investigate phytochemicals, antioxidant properties of the extracts prepared from Tuckeroo fruits and further test their potential anticancer properties using 10 different cancer cell lines.

## Materials and methods

### Plant materials

Ripe fruits of the Tuckeroo (*Cupaniopsis anacardioides*) were collected from Terrigal Beach (33° 26ʹ 52.8396ʹʹ S 151° 26ʹ 40.0596ʹʹ E) and Avoca Beach (33° 27′ 54ʹʹ S 151° 26ʹ 6ʹʹ E), New South Wales, Australia in Summer (Tuckeroo fruits’ season). After collection, fruits were immediately taken to the laboratory of the University of Newcastle and freeze-dried using a freeze dryer (FD3 freeze dyer (Thomas Australia Pty. Ltd., Seven Hills, NSW, Australia). The dried fruits were then ground into powder (less than 1.4 mm in particle size) using a blender (John Morris Scientific, Chatswood, NSW, Australia) and were preserved at – 20 °C for further analysis.

### Preparation of crude *C. anacardioides* extract and its fractions

**Fig. 1:**
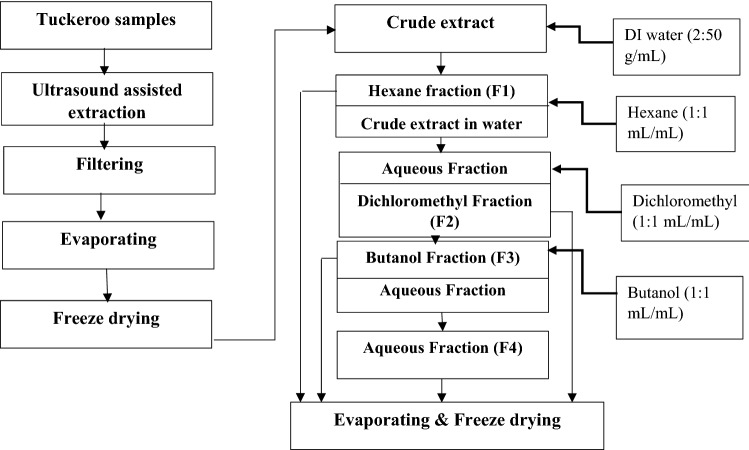
Sample preparation diagram

The crude extract and its fractions were prepared as shown in Fig. 1. The crude extract was obtained by extraction under optimal ultrasonic extraction conditions described in our previous study (Pham et al. 2019). Dried fruit powder (5 g) was extracted in 100 mL acetone 50% using an ultrasonic bath (Soniclean, 220 V, 50 Hz and 250 W, Soniclean Pty Ltd., Thebarton, Australia) set at 150 W, 40 °C for 40 min. After extraction, the extract was centrifuged at 4000 rpm for 10 min at 5 °C (Centrifuge, Beckman J2-MC, Palo Alto, CA, USA) to remove unwanted particles. The extract was then concentrated at low pressure using an evaporator (Buchi Rotavapor B-480, Buchi, Australia, Noble Park, VIC, Australia) and finally 72 h of freeze-dried at – 80 °C to obtain a crude powdered extract using a FD3 freeze dyer (Thomas Australia Pty. Ltd., Seven Hills, NSW, Australia).

Four sub-fractions, including hexane fraction (F1), dichloromethyl fraction (F2), butanol fraction (F3), and aqueous fraction (F4), were subsequently prepared from the crude powdered extract using a liquid–liquid extraction technique. The crude powdered extract was diluted into deionized water with the ratio of 2:50 (g/mL). Hexane, dichloromethyl and butanol solvents were then applied with 1:1 ratio to the diluted crude extract to obtain F1, F2, F3, and F4, as shown in (Fig. 1). The four fractions were then evaporated and freeze-dried to form dried fractions for further analysis.

### Determination of bioactive compounds

The crude powdered extract and its fractions were re-dissolved in 50% methanol (5 g/100 mL). The extracts were then vortexed and sonicated to ensure solubility. Total phenolic content (TPC) and their secondary metabolites including flavonoids (TFC) and proanthocyanidins (TPro) were determined according to previously described methods (Pham et al. 2017; Škerget et al. 2005). TPC was absorbed at 765 nm, using gallic acid as a standard and its value was expressed as milligrams of gallic acid equivalents per gram of dried sample (mg GAE/g DW). TFC and TPro were measured at 510 and 500 nm, respectively, using catechin as a standard and their values were recorded as milligrams of catechin equivalents per gram of dried extract (mg CAE/g DW).

HPLC analysis was applied for determination of major individual compounds in the crude extract and its fractions. A Shimadzu HPLC system (Shimadzu, Tokyo, Japan) equipped with a column (Prodigy 5 µ ODS3 100A; 250 × 46 mm 5 µm) and UV detection (UV–Vis detector SPD-20AV) set at 210 and 280 nm was used to isolate the majority of bioactive components. A volume of 50 µL sample was injected into the column, using an auto injector (SIL-20A HT). The flow rate was set at 1 mL/min, and consisted 0.1% (*v*/*v*) formic acid (solvent A) and absolute acetonitrile (solvent B). The gradient was set as follows: 0–30 min, 20% B; 30–55 min, 60% B; 55–65 min, 100% B; 65–75 min, 30% B; 75–85 min, 0% B and 85–90 min, 0% B. Gemcitabine 1 mM was used as a standard to quantify major compounds in the crude extract and its fractions (Vuong 2015). The results were expressed as mg of gemcitabine equivalents to gram of dried extract (mg GCE/g).

### Determination of the antioxidant activities

To fully reflect the antioxidant capacity from Tuckeroo crude extract and its fractions, four different assays were used, including the DPPH radical scavenging assay, ABTS radical scavenging assay, ferric reducing antioxidant power (FRAP), and cupric-reducing antioxidant capacity (CUPRAC) assays.

DPPH (2, 2-diphenyl-1-picrylhydrazyl) was assessed based on the method as described by Brand-Williams et al. (1995). ABTS [2, 2′-azinobis-(3-ethylbenzothiazoline-6-sulfonic acid)] was determined according to the previous method reported by Thaipong et al. (2006). The FRAP assay was used based on a previous method (Benzie and Strain 1996). CUPRAC was assessed according to Apak et al. (2004). The absorbance was measured at 515, 734, 593, and 450 nm, respectively, using an UV spectrophotometer (Cary 50 Bio Varian, Australia). Trolox was used as a standard curve and the results were expressed as mg of trolox equivalents per g of dried sample (mg TE/g DS).

### Growth inhibition

Cytotoxicity of the Tuckeroo extracts and their semi-purified fractions were assessed in vitro throughout a panel of cancer cell lines including HT29 (colon); U87, SJ-G2 (glioblastoma); MCF-7 (Breast); A2780 (ovarian); H460 (Hsu et al. 2003); A431 (Raskin et al.); Du145 (Gundem et al. 2015); BE2-C (neuroblastoma); MIA PaCa-2 (pancreas); and one non-tumour-derived normal breast cell line (MCF10A). The 3-(4,5-dimethylthiazol-2-yl)-2,5-diphenyltetrazolium bromide (MTT) assay was applied as previously described by Vuong et al. (2015). All tested cells were initial seeded in the 96-well plates with a density of 2500 to 4000 cells per well. Then, they were incubated for 24 h to reach the logarithmic growth. The cytotoxicity of the Tuckeroo extract and its fractions at the concentration of 100 µg/mL were assessed after 72 h of incubation, using MTT assay. Growth inhibition was measured at 540 nm by the optical density differences between these values on day 0 and at the end of treatment.

### Statistical analysis

All experiments were conducted in triplicate. SPSS statistical software (IBM SPSS Statistics 25) was employed, using independent samples t test, one-way ANOVA, and Duncan’s post hoc test to compare the means. Differences between the mean levels were taken to be statistically significant at *p* < 0.05.

## Results and discussion

### Total phenolic content, flavonoids, and proanthocyanidins

**Fig. 2:**
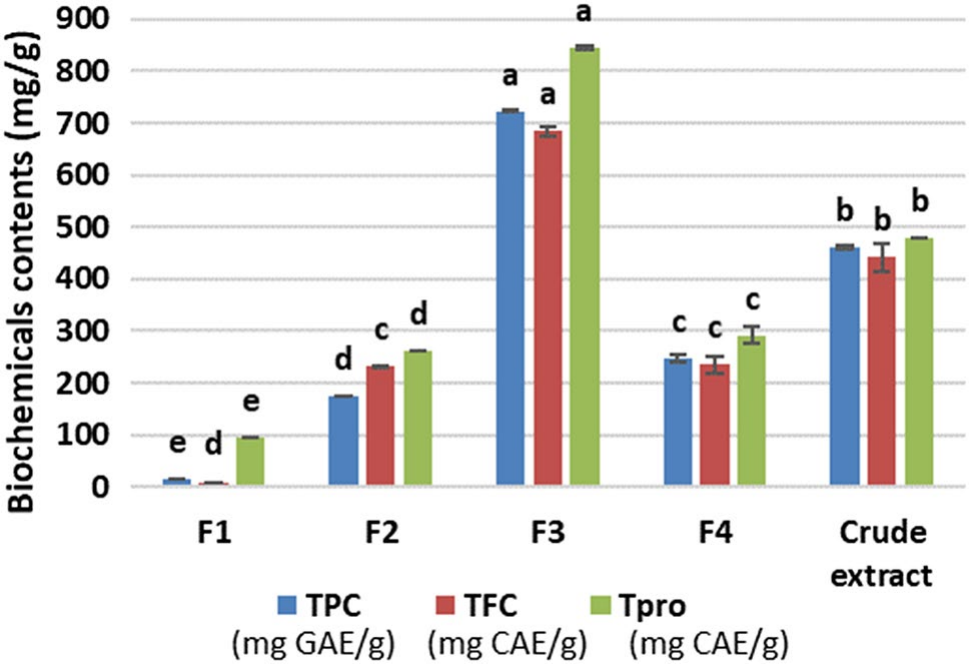
Total phenolic, flavonoid, and proanthocyanidin content from of the Tuckeroo crude extract and its fractions. Data are means ± standard deviations. The different letters on the top of the bars indicate a significant difference at *p* < 0.05

The content of phenolic compounds and their secondary metabolites were significantly different in the crude extract and its fractions (Fig. 2). The highest levels of TPC, TFC, and TPro were found in butanol fraction (F3), followed by the crude extract, aqueous fraction (F4), and dichloromethyl fraction (F2). Hexane fraction (F1) had the lowest levels of TPC, TFC, and TPro. Levels of TPC, TFC, and TPro of F3 were approximately twofold higher than those of the crude extract and over tenfold higher than those of F1. Our findings were similar to previous studies, which reported that hexane fraction had the lowest level of phenolic compounds than crude extract and its other fractions. However, our findings revealed that the butanol fraction (F3) had the highest level of phenolic compounds than those of the crude extract and its other fractions. These were different to other studies who found that the crude extract had the highest level of phenolic content (Wijaya et al. 2017), or ethyl acetate and crude extracts had higher phenolic content than butanol extract (Alidadi et al. 2017). These differences can likely be explained by the various polarities of the phenolic compounds in Tuckeroo fruits. This would lead to different levels of phenolic compounds within the different fractions, and the butanol fraction would solubilise most of phenolic compounds, and thus, this fraction contained the highest levels of TPC, TFC, and TPro.

### Bioactive compounds

**Fig. 3:**
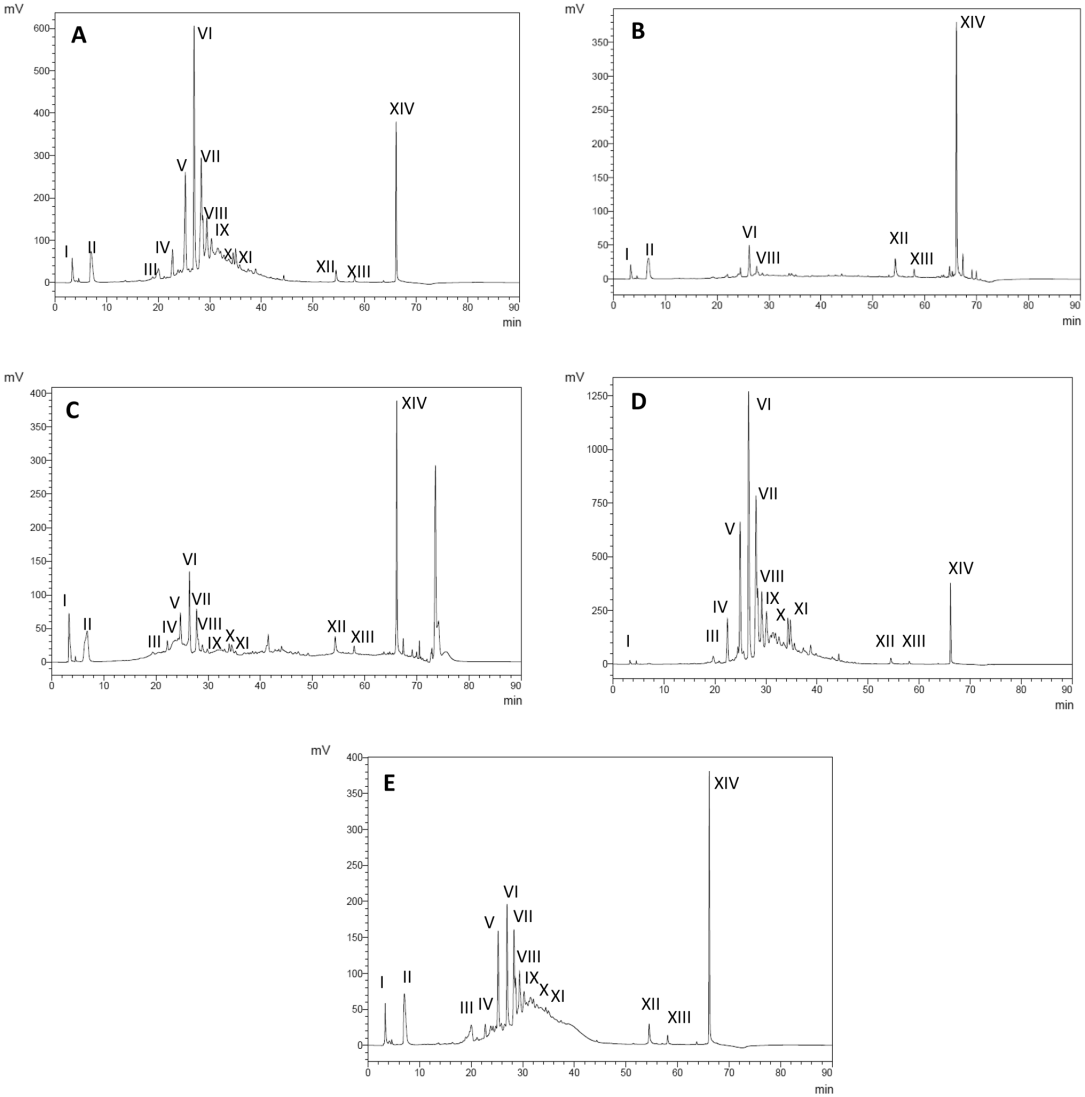
HPLC chromatogram detected at 280 nm for major bioactive compounds of the crude extract (**A**), hexane (**B**), dichloromethyl (**C**), butanol (**D**), and aqueous fraction (**E**)

HPLC analysis was conducted to further isolate and reveal the major bioactive compounds in the Tuckeroo crude extract and its fractions. The results (Fig. 3A, B, C, D, E, Table 1) showed that number and composition of individual phytochemicals differ in the Tuckeroo crude extract when compared to its fractions. Fourteen major components were identified in the Tuckeroo crude extract and its fractions (F2, F3 and F4). There were 7 major compounds observed in the F1 fraction. Total levels of major bioactive compounds were also highest in F3, followed by the crude extract, then F2, and F4. Hexane fraction had the lowest total level of major bioactive compounds. This pattern is similar to that of total phenolic compounds, so it is hypothesised that most major bioactive compounds in the Tuckeroo fruit extract are phenolic compounds. Further studies are recommended to purify and identify these major compounds.

**Table 1:** Concentration of major bioactive compounds of the Tuckeroo crude extract and its fractions

Compounds	Crude extract	F1	F2	F3	F4
I (mg GCE/g)	3.13 ± 0.00^b^	1.22 ± 0.00^d^	4.33 ± 0.00^a^	1.01 ± 0.00^e^	2.49 ± 0.00^c^
II (mg GCE/g)	7.78 ± 0.00^b^	3.45 ± 0.00^c^	9.81 ± 0.00^a^	0.67 ± 0.00^e^	2.97 ± 0.00^d^
III (mg GCE/g)	3.02 ± 0.00^c^	ND	3.87 ± 0.00^b^	3.98 ± 0.00^a^	2.06 ± 0.00^d^
IV (mg GCE/g)	5.72 ± 0.00^c^	ND	30.86 ± 0.00^a^	12.15 ± 0.00^b^	1.44 ± 0.00^d^
V (mg GCE/g)	17.82 ± 0.00^b^	ND	3.21 ± 0.00^d^	34.56 ± 0.00^a^	7.71 ± 0.00^c^
VI (mg GCE/g)	30.16 ± 0.00^b^	3.30 ± 0.00^e^	9.68 ± 0.00^c^	70.6 ± 0.00^a^	8.89 ± 0.00^d^
VII (mg GCE/g)	21.46 ± 0.00^b^	ND	6.29 ± 0.00^d^	45.95 ± 0.00^a^	8.07 ± 0.00^c^
VIII (mg GCE/g)	16.95 ± 0.00^b^	0.96 ± 0.00^e^	3.08 ± 0.00^d^	26.89 ± 0.00^a^	8.75 ± 0.00^c^
IX (mg GCE/g)	14.9 ± 0.00^b^	ND	2.66 ± 0.00^d^	25.45 ± 0.00^a^	5.66 ± 0.00^c^
X (mg GCE/g)	9.03 ± 0.00^b^	ND	2.56 ± 0.00^d^	15.23 ± 0.00^a^	3.46 ± 0.00^c^
XI (mg GCE/g)	5.42 ± 0.00^b^	ND	2.75 ± 0.00^c^	14.30 ± 0.00^a^	2.52 ± 0.00^d^
XII (mg GCE/g)	2.25 ± 0.00^b^	2.12 ± 0.00^d^	6.00 ± 0.00^a^	2.19 ± 0.00^c^	2.25 ± 0.00^b^
XIII (mg GCE/g)	0.90 ± 0.00^c^	ND	3.63 ± 0.00^a^	0.90 ± 0.00^c^	0.91 ± 0.00^b^
XIV (mg GCE/g)	12.71 ± 0.00^d^	13.05 ± 0.00^b^	20.57 ± 0.00^a^	12.74 ± 0.00^c^	12.66 ± 0.00^e^
Total (mg GCE/g)	151.26	24.10	109.31	266.62	69.83

Data are means ± standard deviations. Data in the same row sharing different superscript letters are significantly different at *p* < 0.05. *ND* no data

### Antioxidant activities of the crude extract and its fractions

**Fig. 4:**
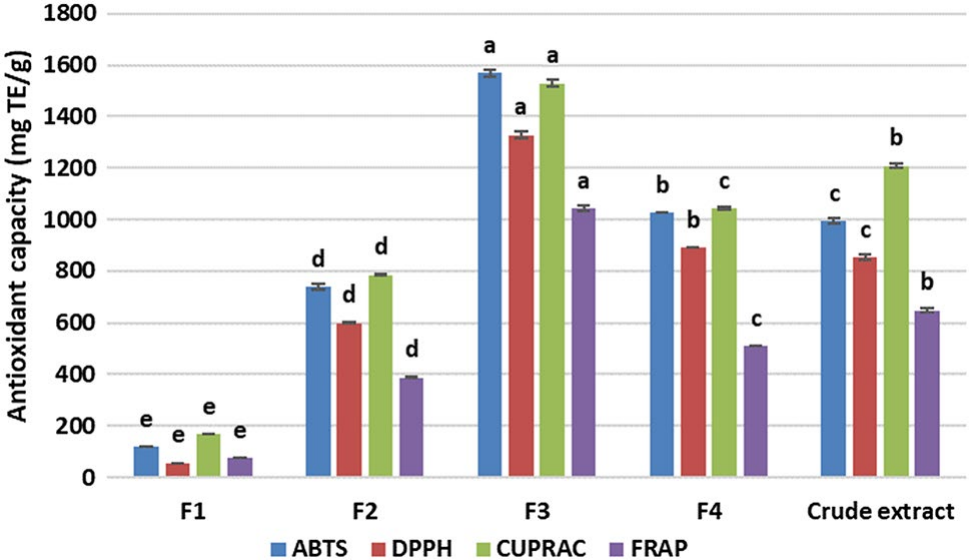
Antioxidant capacity of the Tuckeroo crude extract and its fractions. Data are means ± standard deviations. The different letters on the top of the bars indicate a significant different at *p* < 0.05

Antioxidant activity of the crude extract and its fractions are presented in (Fig. 4). A similar pattern to the total phenolic compounds was observed for the antioxidant activities. The results from the four antioxidant assays showed that F3 had the highest antioxidant activity (ABTS-1570.49 mg TE/g; DPPH-1328.97 mg TE/g; CUPRAC-1529.4 mg TE/g; FRAP-764.7 mg TE/g). This was followed by the crude extract, while the hexane fraction had the lowest levels of antioxidant activity. Previous studies also found that a butanol fraction had the highest level of antioxidant activities as compared to the crude extract and other fractions from *Catharanthus roseus* (L.) G. Don stem ((Pham et al. 2018), and hexane fractions prepared from *Pistasia atlantica* and *Citrus hystrix* peel had the lowest levels of antioxidant activities (Alidadi et al. 2017; Wijaya et al. 2017)). A similar pattern to the phenolics, and variation in antioxidant activities of different fractions can be explained by different levels in phenolic compounds, which are the major antioxidants (antioxidant contributors) in the Tuckeroo extracts (Pham et al. 2017). Plant polyphenols have been known as antioxidants, they have also been reported to be potential anticancer substances and antioxidant activity have been linked with anticancer properties (Grigalius and Petrikaite 2017). Therefore, only the butanol fraction (F3), crude extract, and aqueous fraction (F4), which had potent antioxidant activities, were applied for further testing of their cytotoxicity.

### Anticancer activities

The proportions of cell growth inhibition of the Tuckeroo extract and its butanol and aqueous fractions are shown in Table 2. The results indicated that, except for the cell Du145—prostate cancer cell line, the crude extract (100 µg/mL) could inhibit growth from 33 to 100% for the tested cancer cell lines. Whereas, with the same concentration, F3 could inhibit 27 to > 100% and F4 could inhibit 3 to > 100%. F3 possessed the higher cytotoxicity and growth inhibitory activity in comparison with the crude extract, and F4 within almost tested cancer cell lines, except for breast (MCF-7) and pancreas (MIA PaCa-2) cells, providing an illustration for a good correlation between phytochemicals, antioxidant properties, and anticancer activity. These findings were in agreement with the investigation on *C. roseus* stems by Pham et al. (2018), which reported that the n-butanol fraction had a stronger cytotoxic activity than aqueous fraction. However, prostate cancer cells were not inhibited under the concentration of 100 µg/mL, while 100% growth inhibition of glioblastoma cancer cell (SJ-G2) was observed. As such, a rigorous elucidation of individual component cytotoxicity should be assessed on these cancer cell lines to obtain a more comprehensive understanding of this promising plant material.

**Table 2:** Cell growth inhibition activity (%) of the Tuckeroo extract and its fractions

Cell line	Cancer cell types	Cell growth inhibition (%)		
		Crude extract	F3	F4
HT29	Colon	47 ± 3^b^	58 ± 2^a^	32 ± 3^c^
U87	Glioblastoma	37 ± 5^a^	42 ± 10^a^	23 ± 4^b^
MCF-7	Breast	33 ± 6^a^	27 ± 8^b^	3 ± 7^c^
A2780	Ovarian	81 ± 1^b^	84 ± 1^a^	69 ± 1^c^
H460	Lung	89 ± 3^b^	94 ± 1^a^	81 ± 2^c^
A431	Skin	74 ± 4^b^	90 ± 4^a^	62 ± 5^c^
Du145	Prostate	< 0	< 0	< 0
BE2-C	Neuroblastoma	80 ± 3^c^	89 ± 2^a^	86 ± 2^b^
SJ-G2	Glioblastoma	> 100^a^	> 100^a^	> 100^a^
MIA PaCa-2	Pancreas	99 ± 1^a^	95 ± 1^b^	99 ± 1^a^
MCF10A	Breast (normal)	22 ± 7^b^	54 ± 6^a^	< 0^c^

Data are means ± standard deviations. Data in the same row sharing different superscript letters are significantly different at *p* < 0.05

Table 3 shows the minimum concentration of the crude extract and its semi-purified fractions (µg/mL) required to inhibit the growth of 50% of cancer cells (GI_50_). The lower the values of the GI_50_, the stronger the anticancer activity of the tested extracts. Data from Table 3 reveal that 28–169 (µg/mL) of F3 inhibited growth by 50% of tested cancer cell lines, followed by 37–196 (µg/mL) of the crude extract and 38→200 (µg/mL) of F4. Whereas, GI_50_ values of *Eucalyptus robusta* were ranged from 74 to > 200 (µg/mL), reported by Bhuyan et al. (2017), indicated weaker cytotoxic activity than observed for the Tuckeroo extracts. Interestingly, the cytotoxic activities of the crude extract and both F3 and F4 were strongest against pancreas cancer cells (MIA PaCa-2). The GI_50_ were the greatest for prostate cancer cells (169, 196, and > 200 µg/mL), which was much higher than that of the other tested cancer cell lines. These data were in line with the percentage of growth inhibition outcomes, which indicated that prostate cancer cells were not inhibited at the concentration of 100 µg/mL. Consequently, identification and isolation phytochemical constituents from these extracts are recommended in future research.

**Table 3:** GI_50_ values

Cell line	Cancer cell types	GI_50_ values (µg/mL)		
		Crude extract	F3	F4
HT29	Colon	107 ± 6^b^	89 ± 2^c^	136 ± 2^a^
U87	Glioblastoma	118 ± 6^b^	111 ± 11^b^	153 ± 9^a^
MCF-7	Breast	130 ± 8^c^	157 ± 22^b^	> 200^a^
A2780	Ovarian	40 ± 2^b^	39 ± 2^b^	53 ± 2^a^
H460	Lung	48 ± 4^b^	44 ± 4^c^	57 ± 4^a^
A431	Skin	74 ± 5^ab^	62 ± 4^c^	80 ± 7^a^
Du145	Prostate	196 ± 18^b^	169 ± 2^c^	> 200^a^
BE2-C	Neuroblastoma	67 ± 3^a^	52 ± 2^c^	62 ± 5^ab^
SJ-G2	Glioblastoma	41 ± 2^a^	38 ± 2^ab^	43 ± 4^a^
MIA PaCa-2	Pancreas	37 ± 1^a^	28 ± 2^b^	38 ± 3^a^
MCF10A	Breast (normal)	140 ± 6^b^	99 ± 7^c^	182 ± 10^a^

Data are means ± standard deviations. Data in the same row sharing different superscript letters are significantly different at *p* < 0.05

## Conclusions

The Tuckeroo (*Cupaniopsis anacardioides*) crude extract and its fractions were shown to be a good natural source of phytochemicals with strong antioxidant properties. Fourteen major bioactive compounds were isolated within the three extracts. The butanol fraction (F3) had the greatest levels of total bioactive compounds with strongest antioxidant activity. This fraction also had better anticancer capacity. Therefore, future studies are recommended to purify and identify the major bioactive compounds from the butanol fraction of the Tuckeroo extract and further test their anticancer properties.
